# Positive surgical margin following radical nephrectomy is an independent predictor of local recurrence and disease-specific survival

**DOI:** 10.1186/s12957-017-1257-6

**Published:** 2017-11-02

**Authors:** Yasmin Abu-Ghanem, Jacob Ramon, Raanan Berger, Issac Kaver, Edi Fridman, Raya Leibowitz-Amit, Zohar A. Dotan

**Affiliations:** 1 Department of Urology, Sheba Medical Center, Ramat Gan, Israel; 2 Department of Oncology, Sheba Medical Center, Ramat Gan, Israel; 3 Department of Pathology, Sheba Medical Center, Ramat Gan, Israel; 4 Sackler Faculty of Medicine, Tel Aviv University, Tel Aviv, Israel

**Keywords:** Positive surgical margins, Radical nephrectomy, Renal cell carcinoma, Recurrence, progression and overall mortality, Disease-specific mortality

## Abstract

**Background:**

Positive surgical margins (PSM) are recognized as an adverse prognostic sign and are often associated with higher rates of local and systemic disease recurrence. The data regarding the oncological outcome for PSM following radical nephrectomy (RN) is limited. We examined the predictive factors for PSM and its influence on survival and site of recurrence in patients treated with RN for renal cell carcinoma (RCC).

**Methods:**

Clinical, pathologic and follow-up data on 714 patients undergoing RN for kidney cancer were analyzed. Secondary analysis included 44 patients with metastatic RCC upon diagnosis who underwent cytoreductive nephrectomy (CRN). Univariate and multivariable logistic regression models were fit to determine clinicopathologic features associated with PSM. A Cox proportional-hazards regression model was used to test the independent effects of clinical and pathologic variables on survival.

**Results:**

PSM was documented in 17 cases (2.4%). PSM were associated with tumour size, advanced pathologic stage (pT3 vs. ≤ pT2) and presence of necrosis. On multivariate analysis, cancer-specific survival (CSS) was associated with tumour stage, size, presence of necrosis and PSM. PSM was also associated with local recurrence but not distant metastasis or overall survival (OS). CSS and OS were comparable between the PSM and metastatic RCC groups, but significantly lower than the negative margin group.

**Conclusions:**

The prevalence of PSM following RN is rare. Pathological data, including advanced stage (> pT2), tumour necrosis and tumour size, are associated with the presence of PSM. PSM is associated with tumour recurrence and CSS. Patients with PSM are a potential group for adjuvant therapy or for more careful and thorough follow-up following surgery.

## Background

Positive surgical margins (PSM) are uniformly considered an adverse outcome associated with incomplete cancer removal and are often allied with increased risk of local or distant recurrence [1–3].

The management of patients with PSM remains a challenge in renal cell carcinoma (RCC) patients, with controversy persisting over the need for more rigorous follow-up or for immediate adjunctive therapy. Over the past years, many studies have investigated the outcomes of patients reported to have a PSM following partial nephrectomy (PN). However, a consensus has yet to be reached on the prognostic significance and optimal management of these patients, in comparison to patients with negative surgical margins [4, 5]. During the last decade, PN has become the standard of care for small exophytic, and favourably located renal tumours, based on its provision of equivalent cancer control and better preservation of long-term renal function. However, radical nephrectomy (RN) remains a commonly performed surgery, primarily in the cases in which partial resection is not feasible due to unfavourable tumour location or in patients with locally advanced tumour growth [6]. The data regarding the oncological outcome for PSM following radical nephrectomy is limited.

In the current study, we examined the potential predictors of PSM following RN and investigated long-term oncological outcomes in cases of PSM.

## Methods

Data included all patients who underwent elective radical nephrectomy for renal masses between 1988 and 2013. Patient demographics and surgical details were collected retrospectively following an approval given by our Institutional Review Board. Informed consent was impossible or impracticable to obtain for such research. Research was done only after consideration and approval of a research ethics committee.

Clinical variables recorded included age, gender and co-morbidities. Tumour-related variables included tumour size, side and multifocality. Surgical variables included type of operation (i.e. open or laparoscopic). Pathological variables included capsular invasion, vascular invasion (including renal vein and inferior vena cava), renal pelvis invasion, perinephric fat extension and tumour necrosis. Tumour stage was coded as a dichotomous variable, pT2 or lower vs. pT3. Data pertaining to tumour location or percent parenchyma involvement were unavailable for the majority of patients and were not included.

**Fig. 1:**
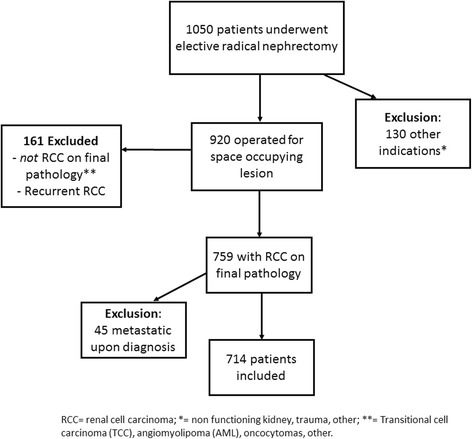
Enrolment and eligibility of subjects who underwent radical nephrectomy between the years 1988 and 2013.

Patients with benign histology (including metanephric adenomas, angiomyolipomas and oncocytomas) were excluded from this analysis. Additional exclusion criteria included patients with malignant tumours other than renal cell carcinoma (urothelial cell carcinoma, sarcoma, neuroendocrine tumour, squamous cell carcinoma, leiomyoma and liposarcoma) or metastatic disease upon diagnosis (Fig. 1). Surgical margin status from the radical nephrectomy was recorded as positive or negative based on macroscopic and microscopic examination of the radical nephrectomy specimen. In all cases, the renal vein, renal vein margin and all other margins were examined grossly and sampled for microscopic examination if suspected of involvement. The interface between tumour and perinephric fat was sampled in all cases to evaluate perinephric fat invasion [7]. PSM were identified from pathology reports (determined by specialized pathologists), using the standard pathology criteria that define a PSM by either extension of tumour to the inked surface of the resected specimen on final pathology or evidence of tumour thrombus invasion into the segmental venous branch, renal vein or inferior vena cava. Tumour size was determined by measuring the maximal diameter of the tumour at pathological examination. Follow-up was conducted according to the standard clinical practice at our institution. In general, follow-up consisted of physical examination, chest radiographs and kidney imaging every 6 to 12 months during the first 5 years and annually thereafter. Patients with PSM on final pathology were observed at similar intervals with serial imaging. None of the patients was treated with immediate adjuvant therapy. Metastatic progression was defined as unequivocal imaging findings indicative of distant organ involvement with or without a confirmatory diagnostic biopsy (based on the discretion of a multi-disciplinary team).

### Statistical analysis

Our main aim was to assess the risk factors for PSM and evaluate cancer control. Univariate and multivariable logistic regression analyses were used to determine features associated with PSM.

Outcomes measured included recurrence-free survival (local and distant), cancer-specific survival (CSS) and overall survival (OS). Our secondary aim was to compare the long-term outcome of these two groups to patients who underwent cytoreductive nephrectomy (CRN) for the treatment of metastatic disease. Secondary analysis included 44 patients with metastatic RCC (mRCC) upon diagnosis who underwent RN to improve survival. All patients were operated in our institute during the same study period. mRCC patients were excluded from primary analysis. Survival curves were estimated using the Kaplan–Meier method, and differences were compared with the log-rank test (*P* < 0.05 was considered statistically significant). Cox proportional-hazard regression models were used to evaluate the association of PSM with outcomes, controlling for clinicopathologic variables. A *P* value < 0.05 was considered statistically significant. Statistical analyses were performed using Statistical Package for Social Sciences (SPSS, Version 21.0, Chicago, IL, USA).

## Results

This study included 714 patients. The clinicopathologic demographics for these patients are provided in Table 1. Of 714 patients, 17 (2.4%) had positive surgical margins at RN. Univariate analysis revealed several variables potentially associated with PSM including tumour size (*P* = 0.001), advanced pathologic stage (*P* = 0.001), central location (*P* = 0.01), tumour necrosis (*P* = 0.001) and capsular invasion (*P* = 0.002). Histologic RCC subtype and operative method (laparoscopic vs. open) were not associated with PSM (Table 2). All variables found significantly related to PSM in the univariate analysis were introduced into a multivariable logistic regression analysis. On multivariate analysis, tumour size (*P* = 0.001), presence of necrosis (*P* = 0.002) and advanced tumour stage (*P* = 0.002) remained significantly associated with PSM (Table 3).

**Table 1:** Clinicopathologic demographics of 714 patients included in the study

Variable	No. (%)	IQR
Age (years)	63.1 ± 12.9	55–72
Gender		
Male Female	439 (61) 275 (39)	
Co-morbidities		
HTN DM Hyperlipidemia IHD Smoking	149 (21) 43 (6) 24 (3) 43 (6) 131 (18)	
Central renal lesion	124 (17)	
Tumour size (cm)	6.7 ± 3.1	4.5–8
Operative method		
Open Lap	481 (67) 233 (33)	
Positive surgical margins	17 (2.4)	
Pathology		
Necrotic tumour Capsular invasion	207 (29.0) 132 (19)	
Tumour stage		
< T2 > T3	440 (62) 274 (38)	
Nuclear grade		
1 2 3 4	50 (7) 335 (47) 250 (35) 78 (11)	
RCC type		
Clear cell Chromophobe Papillary Other Unknown	496 (69) 44 (6) 70 (10) 14 (2) 90 (13)	

Values in parentheses are percentages; continuous variables are presented as (mean ± sd)

*Abbreviations: RCC* renal cell carcinoma

**Table 2:** Univariate analysis of predictive factors for positive surgical margins among patients undergoing radical nephrectomy for renal masses

Variable	No PSM, *n =* 697	PSM, *n =* 17	*P* value
Age	63.0 ± 12.8	66.7 ± 15	0.43
Gender			0.82
Male Female	429 (61) 268 (39)	10 (59) 8 (41)	
Co-morbidities			
HTN DM Hyperlipidemia IHD Smoking	142 (20) 42 (6) 24 (3) 40 (6) 128 (18)	7 (41) 1 (6) 0 (0) 3 (15) 3 (18)	0.04 0.98 0.44 0.04 0.94
Central renal lesion	117 (16.8)	7 (41.2)	0.01
Tumour size			0.01
< 7 cm > 7 cm	419 (60.1) 278 (39.9)	6 (35) 11 (65)	
Nuclear grade			0.08
1 2 3 4	50 (7) 325 (47) 243 (35) 78 (11)	0 (0) 10 (59) 7 (41) 0 (0)	
Operative method			0.6
Open Lap	446 (67) 231 (33)	15 (88) 2 (12)	
Pathology			
Necrotic tumour Capsular invasion	193 (28) 124 (18)	14 (82) 8 (47)	0.001 0.002
Tumour stage			0.001
< T2 > T3	440 (63) 257 (37)	0 (0) 17 (100)	
RCC type			0.26
Clear cell Chromophobe Papillary Other	483 (80) 44 (7) 66 (11) 14 (2)	13 (76) 0 (0) 4 (24) 0 (0)	

Values in parentheses are percentages; continuous variables are presented as (mean ± sd)

*Abbreviations: PSM* positive surgical margins*,* RCC renal cell carcinoma

**Table 3:** Multivariate analysis of the statistically significant predictive factors for positive surgical margins among patients undergoing radical nephrectomy for renal masses

Variable	HR	95 % CI	*P* value
Tumour size	0.4	0.4–0.5	0.001
Tumour necrosis	5.0	1.8–14	0.002
Tumour stage (pT3–4 compared with pT1–T2)	5.5	1.9–15.9	0.002

*Abbreviations: HR* hazard ratio*, CI* confidence interval

### Survival analysis––univariate

Of the study cohort, 102 (14.3%) were lost to follow-up. Survival analysis was done for the remaining 612. The overall median follow-up was 65 months (IQR 27–120). Fifty-three percent of the patients were followed for more than 5 years, and 24.3% of the patients were followed for 10 years and more. There were 50 local recurrence events, and 92 patients developed metastatic progression. Thirty-six (38.3%) of the patients with metastatic progression had previously experienced a local recurrence. The overall 5- and 10-year freedom from local disease recurrence was 91% (95% CI 90, 92) and 89% (95% CI 91, 93), respectively, and freedom from metastatic progression was 84% (95% CI 82, 86) and 81% (95% CI 79, 83), respectively. PSM was associated with significantly worse 5-year freedom from local recurrence (93% compared with 45%; *P* < 0.001) and 5-year freedom from metastatic progression (85 vs. 32%; *P* < 0.001). Similarly, patients with PSM had adverse 5-year CSS (90 vs. 41%; *P* < 0.001) as well as adverse OS (73 vs. 37%; *P* < 0.001).

### Survival analysis––multivariate

We next assessed the association of PSM with outcome, controlling for patient and tumour-related variables (Table 4). We found that PSM remained associated with significantly increased risks of local tumour recurrence (hazard ratio (HR); 4.8, 95% CI 2–11.6, *P* = 0.01) and death from RCC (HR 2.4; 95% CI 1.1–5.5, *P* = 0.03). PSM did not affect the rate of metastatic progression or all-cause mortality. The last was associated with patient’s age (HR 1.03; 95% CI 1.01–1.04, *P* = 0.003), tumour stage (HR 2.2; 95% CI 1.4–3.5, *P* = 0.001) and presence of tumour necrosis (HR 2.0; 95% CI 1.3–2.9, *P* = 0.001). Tumour necrosis (HR 2.2; 95% CI 1.2–4.0, *P* = 0.01) was also associated with increased risks of death from RCC along with advanced pathologic tumour stage (HR 4.2; 95% CI 1.9–9.1, *P* = 0.001) and size (HR 1.1; 95% CI 1.02–1.2, *P* = 0.01).

**Table 4:** Multivariate analysis of factors associated with tumour recurrence, death from renal cancer and all-cause mortality following radical nephrectomy

Variable	Local recurrence			Metastatic progression		
	HR	95% CI	*P* value	HR	95% CI	*P* value
Age	0.99	0.97–1.01	0.3	0.99	0.97–1.0	0.2
Gender	0.75	0.4–1.4	0.4	1.0	0.6–1.6	0.99
Tumour size	1.1	0.99–1.2	0.06	1.1	1.0–1.1	0.03
Central renal lesion	1.0	0.5–2.2	0.9	0.97	0.5–1.8	0.92
Positive surgical margins	4.8	2–11.6	0.001	2.1	0.96–4.4	0.06
Pathology						
Necrotic tumour Capsular invasion	1.1 2.1	0.6–2.2 1.1–4.2	0.7 0.03	2.3 1.4	1.3–3.8 0.8–2.4	0.001 0.2
Tumour stage (pT3–4 compared with pT1-T2)	3.1	1.4–7	0.005	3.6	2.0–6.5	0.001
Variable	All-cause mortality			Disease-specific mortality		
	HR	95% CI	*P* value	HR	95% CI	*P* value
Age	1.03	1.01–1.04	0.003	1.0	0.98–1.02	0.99
Gender	0.8	0.6–1.2	0.3	0.9	0.5–1.6	0.6
Tumour size	1.05	0.99–1.1	0.1	1.1	1.02–1.2	0.01
Central renal lesion	0.7	0.4–1.2	0.3	0.7	0.3–1.5	0.37
Positive surgical margins	1.3	0.6–2.9	0.5	2.4	1.1–5.5	0.03
Pathology						
Necrotic tumour Capsular invasion	2.0 1.3	1.3–2.9 0.8–1.9	0.001 0.2	2.2 1.9	1.2–4.0 1.0–3.5	0.01 0.05
Tumour stage (pT3–T4 compared with pT1–T2)	2.2	1.4–3.5	0.001	4.2	1.9–9.1	0.001

### PSM vs. metastatic disease

Given the poor prognosis of patients with PSM, we next examined whether their oncologic outcomes match those with metastatic disease upon diagnosis (undergoing cytoreductive surgery). Forty-five patients with metastatic RCC who underwent RN were included. All patients in this group had been excluded from the previous analysis.

The subgroup analysis included three groups: non-metastatic, no PSM (*n* = 697), non-metastatic, PSM (*n* = 17) and metastatic (*n* = 45).

**Fig. 2:**
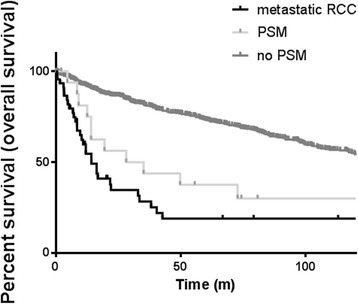
Overall survival curves according to margin status and presence of metastasis. Survival of negative and positive margin patients is significantly different from each other and from patients with metastatic disease (log-rank test, *P* = 0.001).

**Fig. 3:**
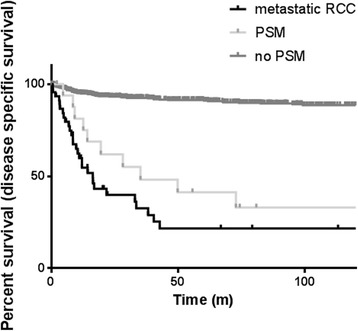
Disease-specific survival curves according to margin status and presence of metastasis. Survival of negative margin patients is significantly different from patients with metastatic disease (log-rank test, *P* = 0.001). Survival of positive margin and metastasis patients is similar (*P* = 0.13).

According to the Kaplan–Meier analysis, median OS was significantly higher in the negative surgical margin group in comparison to both PSM (154 vs. 31.6 months, HR 2.8, 95% CI 2.1 to 14.7, *P* < 0.01) and metastatic group (154 vs.14.3 months, HR 4, 95% CI 7.9 to 30.3, *P* < 0.01). Yet, no differences were found between the PSM and metastatic groups in terms of OS (Fig. 2). Similarly, CSS was found to be significantly poorer in the metastatic group compared to patients with a negative margin (median CSS 16.3 vs. 175.5 months, respectively; *P* = 0.001). Yet, no differences were observed between the metastatic and the PSM groups (Fig. 3).

## Discussion

The achievement of negative surgical margins has a paramount importance and remains the main goal during oncologic surgery. PSM is recognized as an adverse prognostic sign for disease recurrence, especially in tumours of high malignant potential. The potential unfavourable effect of PSM on disease progression and CSS has been previously described in different types of tumours including prostate, rectal and breast cancer but not for renal cancer [8–12].

In recent years, large series have analyzed the preoperative risk factors for PSM after PN. Several factors have been advocated as predictors, such as older age, tumour size, location, pathological stage, Fuhrman grade and indication (elective vs. imperative) [5, 13–15]. Correspondingly, conflicting evidence has accumulated on whether PSM is also a significant risk factor for disease progression, while large-scale studies like Yossepowitch et al., Lopez-Costea et al. or Antic et al. suggest no correlation between PSM and local recurrence or distant progression [14, 16, 17]. Other investigators such as Kwon et al. and Bernhard et al. [18, 19] identified PSM as an independent risk factor for tumour recurrence in the setting of partial nephrectomy, though CSS and OS were not affected by margin status. Yet, despite this discordance, the main implication regarding disease recurrence rate for PSM in partial nephrectomy is the postoperative management. In lieu of the adverse outcome associated with PSM in other solid tumours, patients with PSM should be offered all therapeutic options including radical nephrectomy, repeat PN, energy ablation and vigilant observation [4, 20–22].

Considering the conclusions outlined, it indicates that although the prognostic impact of PSM after PN is ambiguous, these patients should still be closely monitored. Hence, the arising question is whether this conclusion can be applied for patients who undergo radical nephrectomy. To date, PN has been considered the standard of care for the treatment of most renal tumours, with long-term oncologic results equivalent to those of RN. However, PN is unsuitable in some patients with localized RCC due to locally advanced tumour growth or unfeasible because of unfavourable tumour location or significant deterioration in patient health [6]. Thus, the predictive factors for PSM following RN and its implication on patients’ follow-up are a matter of interest. In the current study, positive margins were associated with pathologic stage (≤ pT2 vs. pT3), tumour size and tumour necrosis. Increased pathological stage is associated with positive surgical margin in other malignancies treated surgically such as prostate, bladder and oral cavity cancer [23–25]. According to the current paper, we have demonstrated the association between locally advanced disease (pathological stage (≤ pT2 vs. pT3)) and the presence of PSM. In addition, vascular involvement and tumour necrosis are associated with adverse oncology outcomes in renal cancer and therefore were found to be associated with PSM in addition to pathological stage by multivariate analysis.

In regard to oncologic outcome, previous studies have reported different factors to predict survival after RN, including tumour stage, size, grade and necrosis [7]. An earlier study by Leibovich et al. [26] has reviewed the prediction of progression after RN for patients with clear cell RCC. In his study, Leibovich and colleagues presented a rate of 0.7% PSM. Univariate analysis revealed significant association between PSM and metastasis, yet this factor did not remain significant in a multivariate modeling. Frank et al.[7] presented similar results, including the rate of PSM (0.8%). However, given the small number of cases, Frank and colleagues chose to exclude PSM from further analysis. In the current study, PSM is documented in 17 cases (2.4%). These differences in PSM proportions should be attributed to the pre-disposing tumour properties. Although tumour size was comparable between the studies, nearly 40% of the patients in the current study had pathologic stage of T3 and more, in comparison to 36 and 31% presented by Frank et al. and Leibovich et al., respectively. Moreover, no data is available on the rate of vascular invasion, which is also found to be a significant risk factor for PSM. Finally, both studies conducted analysis only on patients with clear cell RCC, which may have created a selection bias [14].

Our study showed, using multivariable analysis, that the only factor that could predict local recurrence was PSM. In parallel with Leibovich et al., PSM was not associated with metastatic rate, yet tumour necrosis, size and stage could predict metastatic progression. PSM was also associated with CSS along with tumour necrosis and stage, yet not with OS. Further analysis revealed that the effect on survival is so profound that patients with PSM have similar median survival rate to those with metastatic disease upon diagnosis. These results suggest that patients with positive SM, particularly with advanced tumour stage, should be offered more appropriate postoperative surveillance programs, including close monitoring. Currently, we cannot address the role of adjuvant therapy in order to improve the rate of local recurrence, metastasis or cancer-specific survival, due to lack of data to support it. In future, studies that will address the role of adjuvant therapy for high-risk renal cancer following radical nephrectomy should be stratified according to the status of SM.

Our study is not without limitations. First, this study is retrospective, with the entire attendant imprecision associated with the large recollection of data. Second, the number of patients with PSM is small. We acknowledge the fact that 17 patients is a small number; however, giving the potential effect on patients’ outcome and prognosis, analysis of this group, despite its small number, is rather important. To date, the literature relating to this subject is limited, specifically in regard to the association between PSM (following RN) and prognosis. To our knowledge, the current study is the first to directly investigate this association and suggest on its clinical implications. Despite the relatively small number and its limitations, being a retrospective study, we believe that such findings would encourage future randomized clinical trials and will shed the necessary focus on this important entity.

## Conclusion

In this study, we defined the clinical significance of a PSM after RN for RCC. Resection margin appears to be an independent predictor of local recurrence-free and disease-specific survival for all patient subsets. Knowledge of the risk factors for PSM and its influence on disease progression may help clinicians to assess the effects of tumour characteristics on the oncological outcomes following radical nephrectomy, which can be used to improve treatment. The results of this study underscore the significance of histologic resection margin as a prognostic factor after RN.

Future randomized clinical trials are required before we could confirm that the higher incidence rate of PSM does translate into a poorer survival rate.

## Abbreviations

RCC: Renal cell carcinoma
PSM: Positive surgical margins
RN: Radical nephrectomy
PN: Partial nephrectomy
CRN: Cytoreductive nephrectomy
CSS: Cancer-specific survival
OS: Overall survival
